# The “Square Box”: Therapeutic Equivalence as a Foundation of the WHO Model List of Essential Medicines

**DOI:** 10.3389/fphar.2020.578000

**Published:** 2020-09-11

**Authors:** Bernadette Cappello, Lorenzo Moja, Albert Figueras, Nicola Magrini

**Affiliations:** ^1^ Department of Health Products Policy and Standards, World Health Organization, Geneva, Switzerland; ^2^ Department of Pharmacology, Therapeutics and Toxicology, Universitat Autònoma de Barcelona, Barcelona, Spain; ^3^ Italian Medicines Agency, Rome, Italy

**Keywords:** WHO model list of essential medicines, medicines selection, therapeutic equivalence, interchangeability, biologic medicines

## Abstract

Every two years, the World Health Organization (WHO) updates its Model List of Essential Medicines, intended as a guide for countries to adopt or adapt in accordance with local priorities and treatment guidelines, for the development of national essential medicines lists. When more than one therapeutic option is available for a given indication, the WHO Model List often includes a single medicine as representative of a group of equivalent and interchangeable medicines. The representative medicine of that group is listed with an accompanying ‘square box’ symbol. The intended purpose of the square box is to highlight pharmacological classes or groups of medicines for which countries, institutions and health professionals can assume homogeneous therapeutic efficacy and safety and select the most appropriate single medicine based on price, local availability, and acceptability. Though this concept of therapeutic equivalence within a therapeutic class has been endorsed by most authoritative textbooks of pharmacology since *Goodman & Gilman’s The Pharmacological Basis of Therapeutics* and evidence-based guidelines, marketing forces have often made claims on individual drugs to distinguish them beyond relevant differences shown by reliable evidence: this has generated the concept of “me-too drugs” with its double meaning—*i.e.*, market latecomers differing minimally from products preceding them and whose marketing budgets have significant opportunity costs, or medicines which may be useful to substitute for equivalent products in the event of shortages. The square box concept is applied in the context of a comprehensive list: therapeutic equivalence or interchangeability cannot always be easily established. Different interpretations have been applied to different groups of medicines over the 40+ year history of the Model List. This paper presents the concept of the square box, provides key examples and guidance on how square box listings should be practically interpreted in the development and implementation of national essential medicine lists, considers the applicability of a square box listing concept to biologic medicines and proposes that an updated review of the square box concept and listings is warranted.

## Introduction

Markets are filled with thousands of medicines: many are similar pharmacological analogs (so-called “me-too drugs”), offering little, if any, additional clinical benefit in comparison. The expression “me-too” in the field of medicines was first introduced by Goodman in the 1950s and was popularized during the Golden Age of pharmacotherapy, when hundreds of new chemical entities were studied and eventually approved. Describing a medicine as a me-too has a double meaning, that of a “*market latecomer which often differs trivially from earlier products and that the billions of dollars spent marketing these me-too products could be spent in better ways*” (Lee, 2004) but also as medicines which “*may be useful when equivalent drugs can replace each other in the event of shortages*” (Aronson and Green, 2020).

It is thus important to have a mechanism to facilitate the selection of a limited number of essential medicines from the plethora of pharmaceuticals available on the global market. Controlling the number of medicines deemed essential will deliver both healthcare and economic advantages: to facilitate rational prescribing and use by providing more focused information, to enable better value procurement through tendering and competition leading to lower costs for individuals and health systems and to improve access (Hogerzeil, 2004).

The WHO Model List of Essential Medicines was first published in 1977 and has since been recognized as a revolution in public health; introducing the notion that some medicines are more important than others ('t Hoen et al., 2014). WHO defines essential medicines as those that satisfy the priority health care needs of the population, which are intended to be available in functioning health systems at all times in adequate amounts, in appropriate dosage forms, with assured quality, and at prices individuals and the community can afford (WHO, 2011). Medicines are added to or removed from the Model List on the advice of the WHO Expert Committee on Selection and Use of Essential Medicines, an independent, multidisciplinary group of medical and pharmaceutical experts responsible for reviewing medicines and making recommendations to the WHO Director-General. The Expert Committee is convened every two years to evaluate applications proposing additions, deletions, and changes to medicines on the Model List. It is required to make its recommendations having given due regard to disease prevalence and public health relevance, evidence of clinical efficacy and safety, and with consideration of comparative cost and cost-effectiveness (WHO, 2020a).

The Model List is intended as a guide and reference standard for countries for the development of national and institutional essential medicine lists that together with other medicines’ policy actions, empower countries to progress towards universal health coverage and affordable access to safe, effective, and quality-assured essential medicines and health products.

Establishing a limited list of essential medicines is particularly important in low- and middle-income countries, where total health expenditure is more limited and medicine expenditure constitutes a larger proportion of total health expenditure, compared to high income countries (Lu et al., 2011). Investments in medicines should pay worthwhile returns in terms of additional clinical benefit and deliver value for money. In low-, middle-, and high-income countries alike, the costs of many medicines are becoming prohibitive, and policies to improve the efficiency of pharmaceutical spending are increasingly important (OECD, 2010). Square box listings build on the fundamental principal of limited selection. They are intended to give countries scope to select a medicine from within a pharmacological class that best suits local needs based on availability and resources (WHO, 2020a).

Against this background, countries, irrespective of income level, are creating or updating national essential medicines lists, guided by the work done by WHO at the global level. However, countries frequently face uncertainties when undertaking national essential medicine selection, including how to interpret square box listings on the Model List, and determine the alternatives that can be considered therapeutically equivalent. Providing more explicit information on therapeutically equivalent medicines within the square box listings on the Model List can serve to address these uncertainties, better informing and supporting national decision-making.

The aim of this review is to provide guidance for decision-makers to interpret square box listings on the WHO Model List in developing and implementing national essential medicine lists. It also considers the applicability of square box listings to biologic and biosimilar medicines as a mechanism to stimulate competition, reduce cost, and increase access to these therapies.

## History of the Square Box Concept

The concept of a single medicine being included on the Model List as a representative of a broader group of clinically or therapeutically equivalent alternatives has been in place since the first Model List was published in 1977. Listed medicines to which this early concept applied were accompanied by an explanatory note stating “*Listed as an example of this therapeutic category: choose cheapest effective drug product acceptable”* (WHO, 1977). The square box symbol itself (☐) was first introduced into the Model List in 1983 when it was determined that therapeutically equivalent alternatives could be represented by a single medicine, so distinguished by this symbol (WHO, 1983). At that time, this was one of the few special symbols that could be used by typographies around the world, ensuring global understanding of the Model List. By 2002, with a relatively large number of medicines on the Model List with a square box listing (n = 113), the Expert Committee recommended a review of medicines listed with a square box, noting some confusion and inconsistency with regard to the application of the concept and implications for its definition (WHO, 2002). Following consideration of the review in 2003, the Expert Committee made a series of recommendations to retain or remove square boxes and modified the explanatory description of the square box symbol on the Model List:

*“The square box symbol is primarily intended to indicate similar clinical performance within a pharmacological class. The listed medicine should be the example of the class for which there is the best evidence for effectiveness and safety. In some cases, this may be the first medicine that is licensed for marketing; in other instances, subsequently licensed compounds may be safer or more effective. Where there is no difference in terms of efficacy and safety data, the listed medicine should be the one that is generally available at the lowest price, based on international drug price information sources. Therapeutic equivalence is only indicated on the basis of reviews of efficacy and safety and when consistent with WHO clinical guidelines. National lists should not use a similar symbol and should be specific in their final selection, which would depend on local availability and price.”*

This description remains unchanged to date.

The square box is not used to indicate bioequivalence of multisource pharmaceutical products containing the same chemical compound. Bioequivalence of pharmaceutical products is determined by national regulatory authorities and may differ between jurisdictions. The Model List is constructed using the international non-proprietary names of medicines and does not differentiate by proprietary names. Generic substitution between bioequivalent pharmaceutical products is considered acceptable for medicines on the Model List.

## Characteristics of Current Square Box Listings

There are currently 108 entries (involving 93 unique medicines or fixed-dose combinations) on the 2019 Model List that carry a square box symbol (WHO, 2019). These entries appear in the Model List either as “unrestricted” listings, or they are “qualified” by a note specifying the acceptable alternatives. In some cases, there is reference to acceptable alternatives in the technical report of the Expert Committee meeting where specific square box recommendations were made, but the alternatives are not referenced in the list *per se*. An additional 14 entries do not carry a square box but include annotations indicating acceptable alternatives. Square box listings apply to both small molecules and biologic medicines (**Table 1**).

**Table 1:** Square box listings on the 2019 Model List in numbers.

	Number	Examples/comments
Total listings with a square box	108	− 93 unique medicines or fixed-dose combinations − 89 on the core list, 19 on the complementary list
Unrestricted square box*	84	Examples: − simvastatin (representative of statins) − omeprazole (representative of proton pump inhibitors) * some unrestricted listings have reference to acceptable alternatives in the technical report of the meeting where recommendations were made, but these alternatives are not specified in the list.
Qualified square box	24	Examples (specified alternatives): − enoxaparin (nadroparin, dalteparin) − erlotinib (gefitinib, afatinib)
Small molecules	103	Examples (specified alternatives): − bisoprolol (atenolol, metoprolol, carvedilol) − morphine (hydromorphone, oxycodone)
Biological medicines	5	Examples (specified alternatives): − adalimumab [certolizumab pegol, etanercept, golimumab, infliximab (including biosimilars)] − erythropoiesis-stimulating agents [epoetin alfa, beta and theta, darbepoetin alfa, methoxy polyethylene glycol epoetin-beta (including biosimilars)]
Entries without a square box but with named alternatives	14	*e.g.* cycloserine (terizidone), propofol (thiopental), rituximab (quality-assured biosimilars)

### Unrestricted Square Box Listings: All Medicines Are Equal

The majority of square box listings on the 2019 Model List are unrestricted. That is, there is no qualifying note in the list to limit the choice of medicine within the pharmacological class. In these cases, the square box indicates that all medicines within the same pharmacological class can be considered therapeutically equivalent and interchangeable. The representative listed medicine is usually the one for which there is the best or more evidence for effectiveness and safety.

The Model List relies on the Anatomical Therapeutic Chemical (ATC) classification to define medicines within the same pharmacological class. The ATC classification is a 5-level system that classifies medicines according to the anatomical system upon which they act, and their therapeutic, pharmacological and chemical properties. The first level includes fourteen anatomical and pharmacological groups, which are subdivided at the second level into pharmacological or therapeutic groups, at the third and fourth levels into chemical, pharmacological or therapeutic subgroups, with the fifth level being the individual chemical substance (WHO Collaborating Centre for Drug Statistics Methodology, 2020). The ATC classification and the defined daily dose (DDD) assignment are useful tools to identify medicines within pharmacological classes and inform medicine selection decisions at country, institution, or prescriber levels.

Current examples of unrestricted square box listings include proton pump inhibitors (PPIs) for peptic ulcer and gastro-esophageal reflux disease and HMG CoA reductase inhibitors (statins) for hyperlipidemia and prevention of cardiovascular disease, represented on the Model List by omeprazole and simvastatin, respectively. These listings should be interpreted to mean that PPIs and statins have similar clinical performance within their pharmacological classes and represent a group of medicines from which countries can select the most appropriate for their national lists. The ATC classification structures for PPIs and statins are illustrated in **Table 2**. The individual medicines at the fifth ATC level represent options for country-level selection.

**Table 2:** ATC classification structures for proton pump inhibitors and statins.

ATC level	PPIs	ATC code	Statins	ATC code
1: Anatomical main group	Alimentary tract and metabolism	A	Cardiovascular system	C
2: Therapeutic subgroup	Drugs for acid related disorders	A02	Lipid modifying agents	C10
3: Pharmacological subgroup	Drugs for peptic ulcer and gastro-oesophageal reflux disease (GORD)	A02B	Lipid modifying agents, plain	C10A
4: Chemical subgroup	Proton pump inhibitors	A02BC	HMG CoA reductase inhibitors	C10AA
5: Individual chemical substance	omeprazole pantoprazole lansoprazole rabeprazole esomeprazole dexlansoprazole dexrabeprazole lansoprazole, combinations rabeprazole, combinations	A02BC01 A02BC02 A02BC03 A02BC04 A02BC05 A02BC06 A02BC07 A02BC53 A02BC54	simvastatin lovastatin pravastatin fluvastatin atorvastatin cerivastatin rosuvastatin pitavastatin	C10AA01 C10AA02 C10AA03 C10AA04 C10AA05 C10AA06 C10AA07 C10AA08

However, there are some instances where unrestricted square box listings have reference to acceptable alternatives in the technical report of the meeting where recommendations were made, without this recommendation being reflected in the list *per se*. This has the disadvantage for users of the Model List of requiring reference to the historical meeting reports. For example, in 2009, intravenous ibuprofen was included in the Model List with a square box for the management of patent ductus arteriosus in preterm infants. In making this recommendation, the Expert Committee recommended that indomethacin was an appropriate alternative, yet this is not explicit in the list (WHO, 2009). Instances such as this give rise to uncertainty for country-level decision makers and warrant revision as qualified square box listings to increase clarity and eliminate inconsistency.

### Qualified Square Box Listings: When Some Medicines Are Better Than Others

Over 20% of square box listings on the 2019 Model Lists are qualified by a note to indicate that acceptable alternatives are limited to specific medicines. These qualifying notes are recommended by the Expert Committee for a variety of reasons: when there is evidence to suggest within-class differences between medicines (*e.g.*, opioid analgesics) or when there is more limited clinical evidence for some medicines in the class. Qualified square box listings more clearly inform and support rational, evidence-based medicine selection decisions at institution or country level, assist with tendering and procurement processes, and tacitly discourage use of unspecified agents, which would be based on untested assumptions about equivalence in terms of efficacy and safety.

For example, the 2015 addition to the Model List of enoxaparin with a qualified square box listing limits alternatives for low-molecular-weight heparins (LMWHs) to nadroparin and dalteparin, based on the supporting evidence (WHO, 2015). Different LMWHs have been directly compared in a limited number of studies, mostly exploring benefits for venous thromboembolism (White and Ginsberg, 2003). Enoxaparin, nadroparin, and dalteparin are the only LMWHs with evidence in the prevention of venous thrombosis after surgery, as well as for treatment of acute coronary syndromes and venous thromboembolism, the indications for which they are included in the EML. The absence of sufficient evidence on the relative efficacy of other agents in this pharmacological class in conditions other than prevention or treatment of venous thrombosis drove this decision by the Expert Committee.

Similarly, in 2017, the Expert Committee recommended the addition of erythropoiesis-stimulating agents to the Model List for treatment of anemia in patients with chronic kidney disease requiring dialysis, with a qualified square box listing, limiting alternatives to epoetin alfa, beta and theta, darbepoetin alfa, and methoxy polyethylene glycol-epoetin beta (WHO, 2017). The Expert Committee further recommended that their respective biosimilars were also acceptable alternatives based on evidence of therapeutic equivalence and safety of switching to biosimilars from the reference products. Peginesatide, a synthetic erythropoiesis-stimulating agent was not included as an accepted alternative due to serious post-marketing safety concerns that led to its withdrawal from the market in several countries (Hermanson et al., 2016).

In some cases, the square box is used to indicate interchangeability, based on therapeutic indication, of medicines with different pharmacological properties but considered to be therapeutically equivalent alternatives. This use of the square box is only seen in circumstances where comprehensive reviews of efficacy and safety support a conclusion of therapeutic equivalence and when use of the medicines is consistent with recognized clinical guidelines. In such circumstances, the listing is always qualified. However, determining therapeutic equivalence in such circumstances is complex. These cases require detailed and comprehensive review of clinical data on comparative effectiveness and safety and are uncommon. For example, methadone for use in opioid substitution therapy, was added to the Model List in 2005 (WHO, 2005). A square box is included with this listing, along with a qualifying note to identify buprenorphine as an alternative. While methadone and buprenorphine differ in their pharmacological properties, they were considered appropriate therapeutic alternatives for use as substitution therapy for opioid dependence based on the available evidence.

## Application of the Square Box Concept to Biologic or Biotherapeutic Medicines

There is increasing availability of biologic medicines on pharmaceutical markets, many of which are associated with a significant budget impact to health systems. Biosimilar medicines are versions of originator products approved by regulatory agencies that can be manufactured after the originator product patent expires. By virtue of their complex, biological production methods, biosimilar medicines cannot be considered identical to their reference counterparts in the same way that generics of small molecule medicines are considered identical to their reference counterparts (Weise et al., 2014). It has also been noted that each batch of a reference biological is not ‘identical’ to previous or subsequent batches—”*as ‘non-identicality’ is a normal feature of biotechnology that has to be controlled by tight specifications of critical product attributes, within current technical and scientific limitations (inherent variability)”* (Schneider, 2013). Indeed, the promotion and use of biosimilars have given rise to concerns and been the subject of intense debates. However, biosimilars are approved following the same standards of pharmaceutical quality, safety, and efficacy that apply to all biologic medicines, and they can reach the same clinical effect in given clinical settings.

Since 2015, the number of biologics on the Model List has increased, raising the question of how the square box concept should apply to biologic and biosimilar medicines. The 2019 Model List includes erythropoiesis-stimulating agents, enoxaparin, human insulin, filgrastim, leuprorelin, pegaspargase and monoclonal antibodies adalimumab, nivolumab, rituximab and trastuzumab (WHO, 2019). Several of these medicines are listed with a square box. The square box introduces the opportunity at country level for biosimilar versions of these medicines to be procured along with the reference products, or as alternatives. It has been applied when a biosimilar has been shown not to have clinically meaningful differences from the reference product in terms of quality, safety, and efficacy and should be considered to be therapeutically equivalent for national or institutional selection and procurement purposes. The availability of alternative biosimilar medicines also creates increased choice for patients and clinicians.

It is expected that with increasing availability of biosimilar medicines, prices will fall, as has been the experience with the prices of small-molecule medicines with the introduction of generics. Cost-efficiencies can potentially be achieved through increased market competition, which facilitates the treatment of a greater number of patients and adds further sources of supply, potentially reducing the likelihood of shortages (NHS England, 2019). However, in 2019, the Expert Committee expressed concern that to date, availability and access to biosimilars of some essential medicines (*e.g.* rituximab) have been limited. To address this, the Expert Committee recommended WHO to consider expanding its medicines’ prequalification program to include biosimilars of medicines listed on the Model List, such that they are routinely evaluated along with the reference counterparts (WHO, 2019).

Switching from a reference biologic product to its biosimilar or between biosimilar medicines remains a matter of debate in clinical practice (Faccin et al., 2016). The issue of interchangeability and switching between therapeutically equivalent biologic and biosimilar medicines is important for wider access and to foster market competition. The stringent regulatory criteria and the need for providing a comprehensive data package have often been claimed as putting unnecessary burden and cost on the development and licensing of biosimilars, thus leading to delay in the availability of alternatives. On the other hand, these criteria are meant to provide a sufficient level of evidence to reduce patients’ and health care professionals’ concerns about the use of biosimilars. Pre-marketing trials and post-marketing drug-utilization data help to consolidate not only the therapeutic equivalence of two products, but also the safety of switching from reference to biosimilar medicines (Ebbers et al., 2012; D’Amore et al., 2016; CADTH, 2017).

Most biosimilars have been approved after switching patients that have been previously treated with either an originator or a biosimilar medicine. Finally, it should be emphasized that many originators have become biosimilars of themselves due to modifications and improvements in their manufacturing processes after their approval and along the product life cycle (Schneider, 2013). A 2011 study analyzed the quality profiles of market-sourced darbepoetin alfa, rituximab, and etanercept between 2007 and 2010, identifying changes in relevant molecular attributes over time. The tested products remained on the market with unaltered labels, indicating that the changes were not expected to be associated with an altered clinical profile and were thus considered acceptable by health authorities (Schiestl et al., 2011).

## Implications for the Model List for the 2021 Update

Square box listings on the Model List were last reviewed in 2003. An updated review is therefore warranted. In 2021, the Expert Committee will be asked to consider a proposal to revise the definition of the square box concept and the related jargon. “Square box” is a term that lacks both clarity and consistency in its use—a critical flaw for any technical term if it is to be globally understood. The Committee will also be asked to review the existing square box listings and determine the specific alternatives for country-level selection. How best to present square box listings to provide the greatest clarity for countries regarding therapeutically equivalent medicines is important to support informed decision-making about pharmacological class effects.

To clearly indicate appropriate alternatives for therapeutic equivalence, applications for new medicines will need to provide evidence for the medicines deemed to provide equivalent clinical benefits. Applications will also be required to consistently and preferentially use the “qualified” square box listing option. Following this approach, the Model List will continue to recommend a representative medicine for classes of medicines where the Expert Committee accepts therapeutic equivalence. Recognized therapeutically equivalent medicines will be included in the listing in a dedicated field, providing more clear information to support countries in their national selection, tendering, and procurement processes.

The release of an electronic version of the Model List in 2020 (the “eEML”) has introduced the ability to easily and clearly indicate the specific therapeutic alternatives recommended for all medicines listed with a square box, effectively allowing all square box listings to be qualified (WHO, 2020b). This will overcome inconsistency in recommendations and address the uncertainty experienced by country-level decision makers in interpreting some listings with a square box symbol in the traditional print version of the list.

Recently, the Model List has adopted inclusion criteria for quality-assured biosimilars of listed biologic medicines in order to support both their therapeutic equivalence and potential interchangeability. Efforts to identify and address the issues and barriers to interchangeability of biologic medicines to improve access and affordability are also warranted. This includes tackling new approaches to develop, license, and monitor biosimilars to improve efficiency, accelerate access, and reduce uncertainties about their use. It is also relevant to explore different types of switching—from an originator product to a biosimilar (or *vice versa*) or between biosimilars—and if there are implications in terms of efficacy, safety or immunogenicity issues. Recommendations issued by the Expert Committee might target which medicines can be identified as alternatives, how the switch can be implemented (*e.g.* prior to the start of a biological treatment or during prolonged treatment) and policies related to interchangeability. For instance, there are multiple levels at which switching can be enacted: by the physician, by the pharmacist, or by the healthcare system (Barbier et al., 2020). Recommendations could clarify cases in which the switch should be under the responsibility of the treating physician, cases in which the patient can safely switch among biologic and biosimilar medicines (*e.g.* insulins), and cases in which the switch is automatic (*i.e.* without consulting the prescriber). Automatic switching might allow for optimal allocation of resources, given the potential or actual associated cost-reductions (Jensen et al., 2020).

## Conclusions

This review aims to clarify the interpretation and practical application of square box listings of essential medicines to better inform decision-making for national essential medicine lists. The Model List continues to evolve with time, with new medicines regularly considered for inclusion to meet changing public health needs. In 1983, quality information about medicines was scarce and its access very limited; four decades later, prescribers face an overflow of information which mixes biased and low-quality studies with relevant evidence. Additionally, a plethora of me-too medicines have entered the market, many of them without showing clear differences in efficacy or safety over the competitors, yet often at higher costs. Finally, the appearance of biologic products and their biosimilars has changed some paradigms of the chemical medicines and worsened the affordability of essential medicines at country level. Therefore, it is timely to perform an updated review of the square box concept, definition and listings at the next update of the Model List in 2021.

Within this panorama, guidance provided by WHO through the Model List on therapeutic equivalence within a pharmacological class and interchangeability of medicines will support countries to make evidence-based informed choices and avoid listing redundant me-too products with the same efficacy and safety profile. In the case of biological products, the inclusion of biosimilars on the Model List as therapeutic alternatives to reference biologics can serve to improve affordability of otherwise expensive treatments being considered for inclusion on national essential medicines lists by stimulating market competition and introducing opportunities for better value procurement. Utilization studies of access and use policies of medicine classes would enable a better understanding of the impact of the therapeutic equivalence and interchangeability, helping countries to better respond to the strategic opportunities and challenges being faced.

## Author Contributions

BC, LM, and NM contributed to the conceptualization of the review. BC, LM, and AF researched and wrote the manuscript. NM was the Secretary of the WHO Expert Committee on Selection and Use of Essential Medicines between 2015 and 2020 and an employee of the World Health Organization, Geneva, Switzerland, at the time of writing of this paper. All authors contributed to the article and approved the submitted version.

## Conflict of Interest

The authors declare that the research was conducted in the absence of any commercial or financial relationships that could be construed as a potential conflict of interest.
